# Neoadjuvant chemotherapy in older patients with gastric cancer undergoing surgery: a population-based cohort study

**DOI:** 10.1007/s10120-023-01404-2

**Published:** 2023-06-07

**Authors:** Kammy Keywani, Alexander B. J. Borgstein, Wietse J. Eshuis, Marieke Pape, Kathelijn S. Versteeg, Sarah Derks, Hanneke W. M. van Laarhoven, Suzanne S. Gisbertz, Rob H. A. Verhoeven, Mark I. van Berge Henegouwen

**Affiliations:** 1 Department of Surgery, Amsterdam UMC Location University of Amsterdam, Meibergdreef 9, Amsterdam, The Netherlands; 2 Cancer Treatment and Quality of Life, Cancer Center Amsterdam, Amsterdam, The Netherlands; 3 Department of Medical Oncology, Amsterdam UMC Location Vrije Universiteit Amsterdam, De Boelelaan 1117, Amsterdam, The Netherlands; 4 Department of Research and Development, Netherlands Comprehensive Cancer Organisation (IKNL), Utrecht, The Netherlands; 5 Oncode Institute, Utrecht, The Netherlands; 6 Department of Medical Oncology, Amsterdam UMC Location University of Amsterdam, Meibergdreef 9, Amsterdam, The Netherlands

**Keywords:** Gastric cancer, Neoadjuvant chemotherapy, Older patients, Survival

## Abstract

**Background:**

In trials evaluating perioperative chemotherapy for gastric cancer, which serve as the basis for treatment guidelines, patients are selected. The generalizability of these trial findings to older patients is uncertain.

**Methods:**

This population-based retrospective cohort study compared the survival outcomes of patients ≥ 75 years with gastric adenocarcinoma treated with or without neoadjuvant chemotherapy between 2015 and 2019. Additionally, the percentage of patients < 75 years and ≥ 75 years who did not proceeded to surgery after receiving neoadjuvant chemotherapy were examined.

**Results:**

A total of 1995 patients, of whom 1249 aged < 75 years and 746 aged ≥ 75 years, were included. In the group of patients ≥ 75 years, 275 patients received neoadjuvant chemotherapy and 471 patients were directly scheduled for gastrectomy. Patients ≥ 75 years treated with or without neoadjuvant chemotherapy differed significantly from one and another in characteristics. Overall survival of patients ≥ 75 years treated with or without neoadjuvant chemotherapy was not significantly different (median 34.9 vs. 32.3 months; *P* = 0.506), also after adjusting for potential confounders (HR 0.87; *P* = 0.263). Of patients ≥ 75 years who received neoadjuvant chemotherapy, 43 (15.6%) did not proceed to surgery compared to 111 (8.9%) patients < 75 years (*P* < 0.001).

**Conclusion:**

Patients ≥ 75 years treated with or without chemotherapy were highly selected, and overall survival was not significantly different between both groups. Nonetheless, the proportion of patients who did not proceed to surgery following neoadjuvant chemotherapy was higher in patients ≥ 75 years compared to patients < 75 years. Therefore, neoadjuvant chemotherapy should be considered with more caution in patients ≥ 75 years, while identifying those who may benefit.

**Supplementary Information:**

The online version contains supplementary material available at 10.1007/s10120-023-01404-2.

## Introduction

Gastric cancer is often diagnosed in older patients; in 2020, around 46% of patients with gastric cancer were older than 75 years [1]. In the Netherlands, between the years 2011 and 2019, over 30% of gastric cancer patients undergoing potentially curative treatment were over the age of 75 years [2]. In addition, a proportional increase in gastric cancer cases among older patients is anticipated as life expectancy increases.

According to the Dutch and international guidelines, curative treatment with the best survival rates consists of radical (R0) gastrectomy combined with perioperative chemotherapy [3, 4]. These guidelines are based on the outcomes of two large trials, the MAGIC trial [5] and FLOT4 trial [6], which both have shown that perioperative chemotherapy leads as part of curative treatment for gastric cancer to better overall survival. However, the clinical trials were predominantly biased towards enrollment of younger patients. The median age in the MAGIC trial was 62 years, with only 20% of all included patients being 70 years or older [5]. In the FLOT4 trial, the median age was 62 years, with only 24% of all patients 70 years or older [6]. In clinical trials patients are carefully selected due to strict inclusion criteria. The external validity of trial results is questionable for older patients, as individuals participating in trials are generally less complex than many patients seen in geriatric clinics [7–9]. Although, when carefully selected, according to the FLOT4 trial mostly patients older than 70 years seem to benefit from FLOT regimen (fluorouracil plus leucovorin, oxaliplatin and docetaxel) over anthracycline-based triplet regimen [6]. Nonetheless, the population-level effect of neoadjuvant chemotherapy on the older population remains unknown.

The incidence of comorbidities is higher in older patients: 72% of male gastrointestinal cancer patients older than 80 years have comorbidities [10]. The higher incidence of comorbidities in these older patients is associated with more adverse events during chemotherapy and surgery [11]. Previous data showed that 29.3% of gastric cancer patients aged ≥ 75 years did not proceed to surgery after neoadjuvant chemotherapy in the Netherlands between 2006 and 2014 [12]. In older patients, receiving neoadjuvant chemotherapy might preclude a surgical resection more frequently because of adverse events and/or loss of functionality, thereby preventing possible curation. Conversely, not receiving neoadjuvant chemotherapy might deny older patients a survival benefit. In clinical practice, the selection of patients for perioperative chemotherapy can present challenges and is commonly determined primarily by the World Health Organization (WHO) performance status and the existence of comorbid conditions.

The primary aim of this population-based retrospective cohort study was to investigate the overall survival following neoadjuvant chemotherapy versus surgery alone for patients ≥ 75 years with primary resectable gastric adenocarcinoma.

## Methods

This study is a population-based retrospective cohort study using data from the Netherlands Cancer Registry (NCR) [13]. The NCR registers all newly diagnosed patients with cancer. New cases are notified via the National Automated Pathology Archive, which sends weekly notifications of all cases of cancer. Additional medical information concerning patient and tumor characteristics is extracted from medical records by certified data managers of the NCR. Survival status is updated on a yearly basis from the Dutch Personal Records Database. At the time of data extraction, survival follow-up was available until 01-02-2021. Information about progression and recurrences are not recorded as standard by the NCR. This study was approved by the Privacy Review Board of the NCR and the scientific committee of the Dutch Upper GI Cancer Group. Individual informed consent from patients was not required, as obtaining the informed consent was waived by the Dutch Law. All procedures followed were in accordance with the ethical standards of the responsible committee on human experimentation and with the ethical standards of the Helsinki Declaration of 1964 and later versions.

### Study population

Eligible were older patients (≥ 75 years), treated with or without neoadjuvant chemotherapy, and younger patients (< 75 years) treated with neoadjuvant chemotherapy, diagnosed with primary resectable gastric adenocarcinoma, clinically staged as cT1–4A/X, any cN, cM0, who were scheduled for a potentially curative gastrectomy between 2015 and 2019. Patients treated with neoadjuvant (chemo)radiotherapy were excluded. Older patients directly scheduled for surgery who did not undergo resection because of metastatic disease or non-resectable tumor detected at the onset of gastrectomy were also excluded. According to the Dutch gastric cancer guideline, these patients did not undergo a staging laparoscopy [3]. Since metastatic peritoneal disease is often detected by staging laparoscopy [14], including these patients would most likely introduce a source of bias in the survival analysis, as patients who were scheduled to receive neoadjuvant chemotherapy did most likely undergo a staging laparoscopy, in which previously occult peritoneal metastases would probably have been detected.

### Staging and treatment

According to the Dutch gastric cancer guideline, pretreatment clinical staging typically consists of endoscopy with diagnostic biopsies, and computer tomography (CT) of the thorax and abdomen in operable patients with cT3–4 or cN + stage tumors [3]. Since 2016, the Dutch gastric guidelines advises all operable patients with ≥ cT3 and/or N + gastric cancer, without signs of metastases or locoregional non-resectability on initial imaging, a staging laparoscopy before neoadjuvant chemotherapy [3]. Treatment with curative intent usually consists of perioperative chemotherapy (according to the MAGIC [5] or FLOT4 [6] trial), followed by restaging with (PET-)CT and a (sub)total gastrectomy with extensive abdominal lymphadenectomy. Patients are usually directly scheduled for gastrectomy in case of obstruction, bleeding, unfitness or wish not to receive neoadjuvant chemotherapy.

### Data selection

The supplied data of the NCR included the following variables: age, sex, American Society of Anesthesiologists (ASA) classification, WHO-performance status, number of comorbidities, year of diagnosis, tumor location, cTNM classification, tumor differentiation, preoperative treatment regimen (type of chemotherapy regimen and whether or not all cycles were completed), pathological response to neoadjuvant treatment, number of patients proceeding to surgery and reasons for not proceeding, resection type, (y)pTNM classification, resection margin, 30-day postoperative complications, length of hospital stay, type of adjuvant treatment, and overall survival. Tumor location was categorized as proximal (cardia, fundus, and corpus), distal (antrum and pylorus), and whole stomach. The seventh TNM staging edition [15] was used for clinical and pathological TNM staging between 2015 and 2016, from 2017 onwards the eight edition of the TNM staging [16] was used.

### Study outcomes

The primary outcome was the overall survival of patients ≥ 75 years who received neoadjuvant chemotherapy and patients ≥ 75 years directly scheduled for gastrectomy. In addition, the primary outcome was stratified in patients ≥ 75 years by chemotherapy regimen (FLOT versus anthracycline-based triplet therapy). Overall survival was defined as the time from diagnosis until death from any cause. The secondary endpoint of the study was the proportion of patients < 75 years and patients ≥ 75 years who did not proceed to surgery after receiving neoadjuvant chemotherapy. This outcome measure was stratified according to different age groups (≥ 80, 75–79, 70–74, and < 70 years). Secondary outcomes additionally included completion of neoadjuvant chemotherapy, R0-resection rate, hospital stay, and 30-day postoperative complications and mortality.

### Statistical analysis

Baseline characteristics were analyzed using descriptive statistics. Results were presented as mean (standard deviation [SD]) for normally distributed variables, median (interquartile range [IQR]) for non-normally distributed variables, and counts (percentage) for categorical variables. Univariate analyses of the two cohorts were compared using independent t-test or Mann–Whitney U test for continuous variables and *x*^2^*-*test or Fisher’s exact test for categorical variables, when appropriate. The Kaplan–Meier method was used to estimate the overall survival and compared between groups using the log-rank test on an intention-to-treat basis. To adjust for the influence of potential confounders on overall survival, the following covariates were added to a multivariable Cox proportional hazard model: age, sex, WHO performance status, number of comorbidities, tumor location, cT-stage, cN-stage, and tumor differentiation. Missing data were not imputed and cases with a missing value on a specific variable were not dropped when calculating *P*-values. Furthermore, propensity score matching was performed to assess the sensitivity of the Cox proportional hazard model. A propensity score on receiving neoadjuvant chemotherapy or upfront surgery was calculated for each older patient through multivariable logistic regression based on the same baseline characteristics as used in the multivariable Cox proportional hazard model. After applying propensity score trimming to exclude extreme propensity scores, a 1:1 nearest-neighbor matching without replacement was used to create two matched groups. After matching, balance was assessed with the standardized mean difference and differences of more than 10% were assumed to represent an inadequate balance. This resulted in a matching with a caliper width of 0.08 of the pooled standard deviation of the logit of the propensity score, in which all items had a standardized mean differences of < 10%. For the propensity score matched group, the Kaplan–Meier method was used to estimate overall survival and compared using the log-rank test and univariate Cox proportional hazard regression on an intention-to-treat basis. For all analyses, a 2-sided *P* < 0.05 was considered statistically significant. Data was analysed using SPSS version 26 (IBM, Armonk, New York, USA).

## Results

### Study populations

**Fig. 1:**
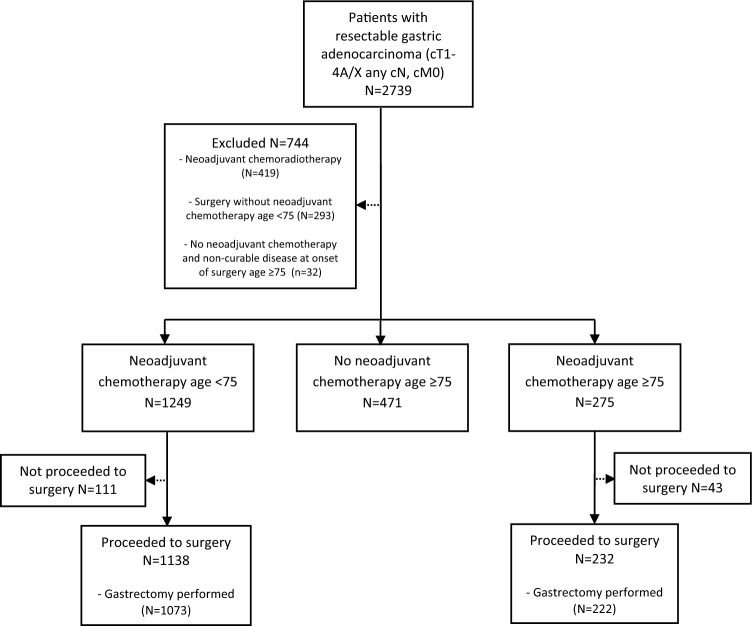
Flowchart of 2739 patients with potentially curable gastric adenocarcinoma

A total of 1995 patients, of whom 1249 (62.6%) aged < 75 years and 746 (37.4%) aged ≥ 75 years, were included in this study (Fig. 1). In the group of 746 patients ≥ 75 years, 275 (36.9%) patients received neoadjuvant chemotherapy and 471 (63.1%) patients were directly scheduled for surgery. In patients ≥ 75 years, those who were directly scheduled for surgery had a higher age (median 80 years [IQR 78–83] vs. 77 years [IQR 76–78]; *P* < 0.001), a greater percentage had WHO performance status ≥ 2 (14.4% vs. 4.7%; *P* < 0.001) and ASA score ≥ 3 (48.8% vs. 28.4%; *P* < 0.001), with lower proportion cT3-T4 tumors (29.1% vs. 8.0%; *P* < 0.001), and more often cN0 disease (70.5% vs. 53.8%; *P* < 0.001), distal tumors (51.8% vs. 36.0%; *P* < 0.001), and poorly differentiated tumors (52.2% vs. 44.4%; *P* < 0.001) compared to patients ≥ 75 years treated with neoadjuvant chemotherapy (Table 1). Baseline characteristics of patients < 75 years and ≥ 75 years treated with neoadjuvant chemotherapy were comparable, except that in patients ≥ 75 years treated with neoadjuvant chemotherapy a higher proportion had ASA score ≥ 3 (28.4% vs. 21.3%; *P* < 0.001), multiple comorbidities (20.0% vs. 11.9%; *P* < 0.001), well to moderate differentiated tumors (34.5% vs. 27.5%; *P* = 0.044), and lower incidence of proximal tumors (45.8% vs. 52.3%; *P* = 0.028). Following the 1-to-1 matching by propensity score in the patient group ≥ 75 years, 169 patients treated with neoadjuvant chemotherapy were matched to 169 patients directly scheduled for surgery. Adequate balance was achieved for all baseline characteristics (Supplementary Table S1).

**Table 1:** Baseline and tumor characteristics of patients < 75 years treated with neoadjuvant chemotherapy and patients ≥ 75 years treated with or without neoadjuvant chemotherapy

Patients characteristics	1	2	3	1 versus 2	2 versus 3
	Neoadjuvant chemotherapy age < 75 (N = 1249)	Neoadjuvant chemotherapy age ≥ 75 (N = 275)	No neoadjuvant chemotherapy age ≥ 75 (N = 471)	*P*-value	*P*-value
Age, median (IQR), years	64 (57–70)	77 (76–78)	80 (78–83)	< 0.001	< 0.001
Sex, male no./total no. (%)	824 (66.0)	172 (62.5)	285 (60.5)	0.280	0.622
WHO performance status, no./total no. (%)				0.348	< 0.001
0	540 (43.2)	103 (37.5)	92 (19.5)		
1	408 (32.7)	96 (34.9)	126 (26.8)		
≥ 2	49 (3.9)	13 (4.7)	68 (14.4)		
Unknown	252 (20.2)	63 (22.9)	185 (39.3)		
ASA classification, no./total no. (%)				< 0.001	< 0.001
1	77 (6.2)	7 (2.5)	6 (1.3)		
2	615 (49.2)	103 (37.5)	195 (41.1)		
≥ 3	266 (21.3)	78 (28.4)	230 (48.8)		
Unknown	291 (23.3)	87 (31.6)	40 (8.5)		
Number of comorbidity categories, no./total no. (%)				< 0.001	0.224
0	633 (50.7)	112 (40.7)	162 (34.4)		
1	360 (28.8)	90 (32.7)	159 (33.8)		
≥ 2	149 (11.9)	55 (20.0)	121 (25.7)		
Unknown	107 (8.6)	18 (6.5)	29 (6.2)		
Year of diagnosis no./total no. (%)				0.414	0.062
2015	255 (20.4)	52 (18.9)	105 (22.3)		
2016	267 (21.4)	48 (17.5)	106 (22.5)		
2017	220 (17.6)	48 (17.5)	92 (19.5)		
2018	266 (21.3)	70 (25.5)	85 (18.0)		
2019	241 (19.3)	57 (20.7)	83 (17.6)		
Tumor location, no./total no. (%)				0.028	< 0.001
Proximal	653 (52.3)	126 (45.8)	164 (34.8)		
Distal	420 (33.6)	99 (36.0)	244 (51.8)		
Whole stomach	156 (12.5)	39 (14.2)	26 (5.5)		
Unknown	20 (1.6)	11 (4.0)	37 (7.9)		
cT-stage, no./total no. (%)				0.320	< 0.001
T1–T2	420 ( 33.6)	105 (38.2)	207 (43.9)		
T3–T4a	657 (52.6)	132 (48.0)	137 (29.1)		
Tx	172 (13.8)	38 (13.8)	127 (27.0)		
cN-stage, no./total no. (%)				0.400	< 0.001
N0	642 (51.4)	148 (53.8)	332 (70.5)		
N1	338 (27.1)	80 (29.1)	94 (20.0)		
≥ N2	215 (17.2)	39 (14.2)	30 (6.4)		
Nx	54 (4.3)	8 (2.9)	15 (3.2)		
Tumor differentiation, no./total no. (%)				0.044	< 0.001
Well-moderate	343 (27.5)	95 (34.5)	174 (36.9)		
Poorly	644 (51.6)	122 (44.4)	246 (52.2)		
Unknown	262 (21.0)	58 (21.1)	51 (10.8)		

*IQR* interquartile range, *WHO* World Health Organization, *ASA* American Society of Anesthesiologists Classification

### Completion of neoadjuvant chemotherapy and proceeding to surgery

A stratification of patients by age categories ≥ 80, 75–79, 70–74, and < 70 years revealed that the median interval between diagnosis and onset of neoadjuvant chemotherapy was 44 (IQR 33–55), 42 (IQR 32–54), and 40 (IQR 31–52), 38 (IQR 29–50) days, respectively (*P* < 0.001) (Table 2). Patients more often received doublet-based chemotherapy in the higher age groups (23.7% of patients aged ≥ 80 years, 19.0% of patients aged 74–79 years, 10.2% of patient aged 70–74 years, and 4.6% of patients aged < 70 years; *P* < 0.001). In the group of patients aged ≥ 80 years who received neoadjuvant chemotherapy, 68.4% patients completed all cycles, compared to 62.4% of patients aged 75–79 years, 64.0% of patients aged 70–74 years, and 77.3% of patients aged < 70 years (*P* < 0.001).

**Table 2:** Neoadjuvant treatment regimen and outcomes by age categories

	Patients < 75 years (N = 1249)		Patients ≥ 75 years (N = 275)		*P*-value*
	age < 70 (N = 916)	70–74 (N = 333)	Age 75–79 (N = 237)	≥ 80 (N = 38)	
Interval between diagnosis and onset of neoadjuvant therapy (days), median (IQR)	38 (29–50)	40 (31–52)	42 (32–54)	44 (33–55)	< 0.001
Type of neoadjuvant therapy, no./total no. (%)					0.175
Chemotherapy	899 (98.1)	331 (99.4)	231 (97.5)	37 (97.4)	
Chemo- and targeted therapy	17 (1.9)	2 (0.6)	6 (2.5)	1 (2.6)	
Neoadjuvant chemotherapy regime, no./total no. (%)					< 0.001
EOX/ECC/EOF/ECF	548 (59.8)	178 (53.5)	108 (45.6)	11 (28.9)	
FOLFOX/CAPOX	42 (4.6)	34 (10.2)	45 (19.0)	9 (23.7)	
FLOT	263 (28.7)	106 (31.8)	65 (27.4)	12 (31.6)	
DOC	18 (2.0)	7 (2.1)	7 (3.0)	0 (0.0)	
Other	45 (4.9)	8 (2.4)	12 (5.1)	6 (15.8)	
Course of neoadjuvant regime no./total no. (%)					< 0.001
Completed all cycles	708 (77.3)	213 (64.0)	148 (62.4)	26 (68.4)	
Reduction in cycles	131 (14.3)	100 (30.0)	71 (30.0)	5 (13.2)	
Unknown	77 (8.4)	20 (6.0)	18 (7.6)	7 (18.4)	
Not proceeded to surgery after neoadjuvant therapy no./total no. (%)	77 (8.4)	34 (10.2)	33 (13.9)	10 (26.3)	< 0.001

	N = 77	N = 34	N = 33	N = 10	
Reasons for not proceeding to surgery no./total no. (%)					0.011
Non-curable disease after restaging	23 (29.9)	7 (20.6)	4 (12.1)	1 (10.0)	
Poor functional status	6 (7.8)	2 (5.9)	10 (30.3)	1 (10.0)	
Patient’s request	4 (5.2)	2 (5.9)	2 (6.1)	0 (0.0)	
Low tumorload	1 (1.3)	0 (0.0)	0 (0.0)	0 (0.0)	
Deceased	2 (2.6)	2 (5.9)	1 (3.0)	0 (0.0)	
Unknown	41 (53.2)	21 (61.8)	16 (48.5)	8 (80.0)	

*IQR* interquartile range, *EOX* epirubicine, oxaliplatin, capecitabine, *ECC* epirubicine, cisplatin, capecitabine, *EOF* epirubicine, oxaliplatin, 5-fluorouracil, *ECF* epirubicine, cisplatin, 5-fluorouracil, *FOLFOX* 5-fluorouracil, oxaliplatin, *CAPOX* capecitabine, oxaliplatin, *FLOT* 5-fluorouracil, leucovorin, oxaliplatin, docetaxel, *DOC* docetaxel, oxaliplatin, capecitabine

*P-value is based on analysis of patients < 75 years (n = 1249) versus patients ≥ 75 years (n = 275)

The proportion of patients who did not proceed to surgery after receiving neoadjuvant chemotherapy varied across different age categories; 26.3% of patients aged ≥ 80 years, 13.9% of patients aged 75–79 years, 10.2% of patients aged 70–74 years, and 8.4% of patients aged < 70 years. Overall, out of the 275 patients ≥ 75 years and 741 patients < 75 years, 43 (15.6%) patients and 111 (8.9%) patients did not proceed to surgery (*P* < 0.001). When stratifying the data by the type of chemotherapy regimen in patients ≥ 75 years, there was no significant difference in the proportion of patients who did not proceed to surgery between those receiving anthracycline-based triplets and those receiving FLOT (12.6% vs. 6.5%; *P* = 0.145) (Supplementary Table S2).

Patients ≥ 75 years who proceeded to surgery differed significantly in characteristics compared to patients ≥ 75 years who did not proceed to surgery; a lower proportion had unknown tumor differentiation (17.2% vs. 41.9%; *P* < 0.001), a higher proportion had clinically unaffected lymph nodes (56.0% vs. 41.9%; *P* = 0.027), a greater percentage received FLOT regimen (31.0% vs. 11.6%; *P* < 0.001), and relative more patients completed all preoperative chemotherapy cycles (68.5% vs. 34.9%; *P* < 0.001) (Supplementary Table S3).

Out of 232 patients ≥ 75 years who received neoadjuvant chemotherapy and proceeded to surgery, 222 (95.7%) patients underwent gastrectomy and 10 (4.3%) patients were diagnosed with non-curable disease during intended gastrectomy and underwent no resection accordingly. Relative more patients ≥ 75 years who received neoadjuvant chemotherapy underwent a total gastrectomy, compared to patients ≥ 75 years who were directly scheduled for surgery (40.1% vs. 26.5%; *P* < 0.001), and more patients had a (y)pT0 (9.0% vs. 0.0%; *P* < 0.001) (Table 3). Postoperative complication rates were comparable between the two groups of patients ≥ 75 years treated with or without neoadjuvant chemotherapy who underwent surgery (27.9% vs. 34.8%; *P* = 0.194), including the length of postoperative hospital stay (median both groups 8 days [IQR 6–11 vs. 6–14]; *P* = 0.097). Regarding surgical mortality, the 30-day postoperative mortality rate was lower in patients ≥ 75 years treated with neoadjuvant chemotherapy compared to patients ≥ 75 years directly scheduled for surgery (3.2% vs. 8.3%; *P* = 0.011).

**Table 3:** Surgical, pathological, and adjuvant treatment details of patients ≥ 75 years who received surgery with or without neoadjuvant chemotherapy

	Neoadjuvant chemotherapy age ≥ 75 (N = 232)	No neoadjuvant chemotherapy age ≥ 75 (N = 471)	*P*-value
Interval between onset of neoadjuvant therapy and surgery (days), median (IQR)	93 (83–105)	–	
Non-curable disease during intended gastrectomy no./total no. (%)	10 (4.3%)	–	
Resection performed	N = 222	N = 471	
Resection type, no./total no. (%)			< 0.001
Total gastrectomy	89 (40.1)	125 (26.5)	
Subtotal gastrectomy	117 (52.7)	331 (70.3)	
Esophagectomy	16 (7.2)	15 (3.2)	
Pathological response to neoadjuvant therapy, no./total no. (%)			
Complete	18 (8.1)	–	
Subtotal	21 (9.5)	–	
Partial	81 (36.5)	–	
None	60 (27.0)	–	
Unknown	42 (18.9)	–	
(y)pT-stage, no./total no. (%)			< 0.001
T0	20 (9.0)	0 (0.0)	
T1	22 (9.9)	94 (20.0)	
T2	41 (18.5)	56 (11.9)	
T3	91 (41.0)	186 (39.5)	
T4	48 (21.6)	132 (28.0)	
Tx	0 (0.0)	3 (0.6)	
(y)pN-stage, no./total no. (%)			0.062
N0	96 (43.2)	175 (37.2)	
N1	54 (24.3)	92 (19.5)	
N2	34 (15.3)	82 (17.4)	
N3	37 (16.7)	114 (24.2)	
Nx	1 (0.5)	8 (1.7)	
Resection margin, no./total no. (%)			0.098
R0	183 (82.4)	388 (82.4)	
R1	18 (8.1)	52 (11.0)	
R2	0 (0.0)	9 (1.9)	
Unknown	21 (9.5)	22 (4.7)	
30-day postoperative complications, no./total no. (%)			0.194
Yes	62 (27.9)	164 (34.8)	
Unknown	38 (17.10)	71 (15.1)	
Type of complications, no./total no. (%)			0.091
Pulmonary	17 (7.7)	20 (4.2)	
Cardiac	2 (0.9)	13 (2.8)	
Trombo-embolic	3 (1.4)	4 (0.8)	
Anastomotic leakage	6 (2.7)	15 (3.2)	
Chyle leakage	3 (1.4)	3 (0.6)	
Wound infection	12 (5.4)	30 (6.4)	
Neurological	4 (1.8)	10 (2.1)	
Multiple	15 (6.8)	69 (14.6)	
Length of hospital stay (days), median (IQR)	8 (6–11)	8 (6–14)	0.097
30-day postoperative mortality no. (%)	7 (3.2)	39 (8.3)	0.011
Adjuvant therapy no./total no. (%)			< 0.001
Yes	88 (39.6)	5 (1.1)	
Type of adjuvant therapy no./total no. (%)			< 0.001
Chemotherapy	84 (95.5)	0 (0.0)	
Chemoradiotherapy	4 (4.5)	5 (100.0)	

IQR, interquartile range

### Overall survival

**Fig. 2:**
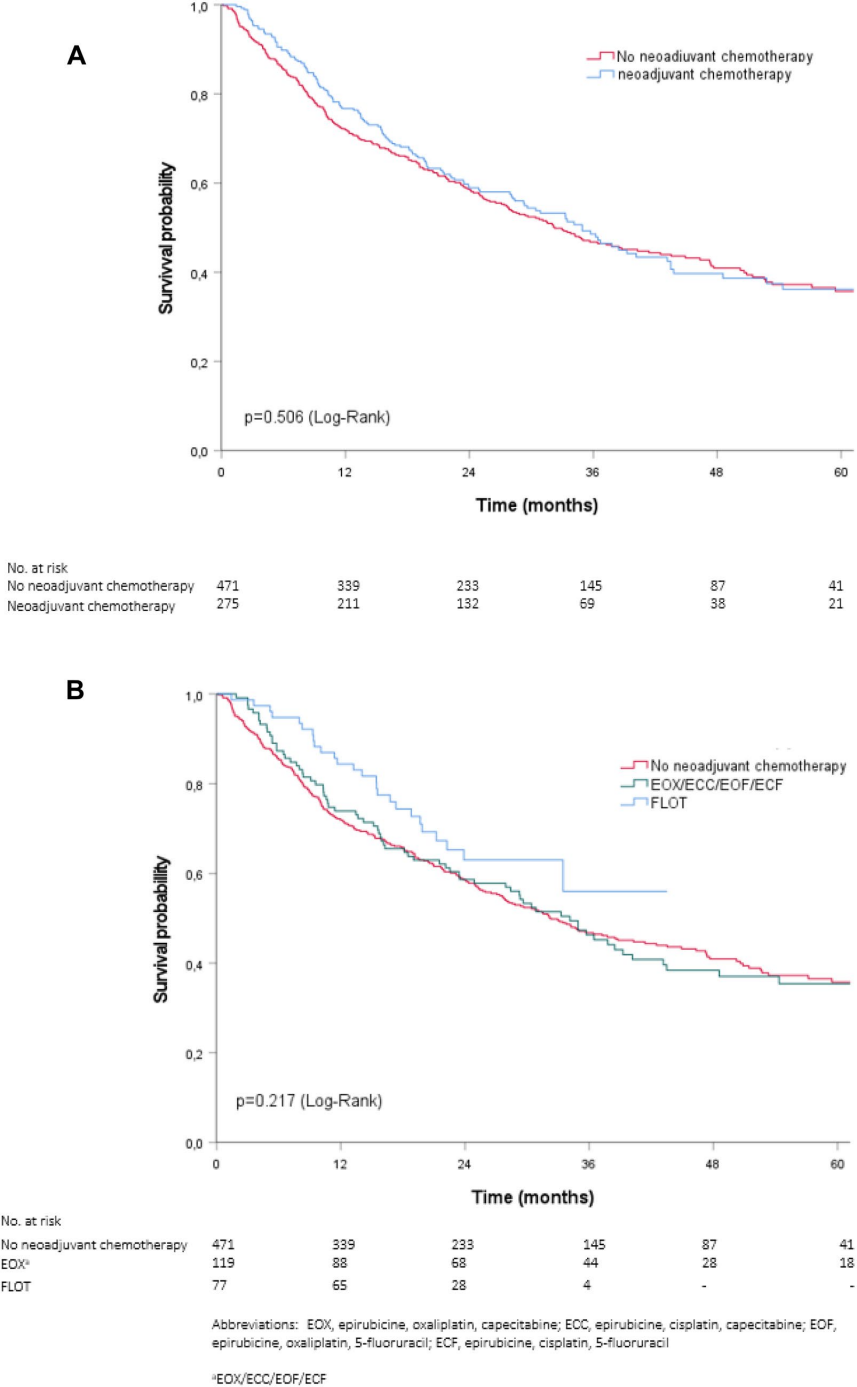
**A** Overall survival of patients ≥ 75 years treated with or without neoadjuvant chemotherapy. **B** Overall survival of patients ≥ 75 years by neoadjuvant treatment regimen

The median overall survival time was 34.9 months (95% confidence interval [CI], 29.2–40.7) for patients ≥ 75 years treated with neoadjuvant chemotherapy and 32.3 months (95% CI 26.3–38.3) for patients ≥ 75 years directly scheduled for surgery (*P* = 0.506) (Fig. 2a and Table 4). The estimated overall survival at 3 and 5 years were 49% and 36% for patients ≥ 75 years treated with neoadjuvant chemotherapy compared to 47% and 36% for patients ≥ 75 years directly scheduled for surgery. The unadjusted mortality risk was similar for patients ≥ 75 years treated with neoadjuvant chemotherapy and patients ≥ 75 years directly scheduled for surgery (HR 0.93; 95% CI 0.76–1.14). Overall survival remained comparable between patients ≥ 75 years treated with neoadjuvant chemotherapy and those directly scheduled for surgery after adjusting for potential confounders (HR 0.87; 95% CI 0.67–1.12). A similar overall survival was found in the sensitivity analysis among the propensity score matched population of patients ≥ 75 years treated with or without neoadjuvant chemotherapy (HR 0.85; 95% CI 0.64–1.14) (Supplementary Table S4).

**Table 4:** Survival of patients ≥ 75 years treated with or without neoadjuvant chemotherapy, and by anthracycline-based triplet therapy and FLOT regimen

	Neoadjuvant chemotherapy age ≥ 75 (N = 275)	No neoadjuvant chemotherapy age ≥ 75 (N = 471)	*P*-value
Overall survival (months), median (95% CI)	34.9 (29.2–40.7)	32.3 (26.3–38.3)	0.506
3-year overall survival %	49	47	
5-year overall survival %	36	36	
Unjadusted hazard ratio (95% CI)	0.93 (0.76–1.14)	1	0.506
Adjusted hazard ratio (95% CI)*	0.87 (0.67–1.12)	1	0.263

	EOX/ECC/EOF/ECF (N = 119)	FLOT (N = 77)	*P*-value
Overall survival (months), median (95% CI)	34.1 (27.1–41.2)	–	0.164
3-year overall survival %	46	56	
5-year overall survival %	35	–	

*EOX* epirubicine, oxaliplatin, capecitabine, *ECC* epirubicine, cisplatin, capecitabine, *EOF* epirubicine, oxaliplatin, 5-fluorouracil, *ECF* epirubicine, cisplatin, 5-fluoruracil, *FOLFOX* 5-fluorouracil, oxaliplatin, *CAPOX* capecitabine, oxaliplatin, *FLOT* 5-fluorouracil, leucovorin, oxaliplatin, docetaxel, *DOC* docetaxel, oxaliplatin, capecitabine

*Adjusted for age, sex, WHO performance status, number of comorbidities, tumor location, cT-stage, cN-stage, and tumor differentiation

Patients ≥ 75 years who were treated with FLOT had similar overall survival (median survival time not reached) as compared to patients ≥ 75 years who received an anthracycline-based triplet regimen [median 34.1 months (95% CI 27.1–41.2, *P* = 0.164) (Fig. 2b and Table 4)]. The estimated 3-year overall survival rates were 56% for patients ≥ 75 years treated with FLOT and 46% for those treated with anthracycline-based triplet therapy. The 5-year overall survival rates could not be estimated for the FLOT group, and for patient ≥ 75 years treated with anthracycline-based triplet therapy 5-year survival rates were 35%.

## Discussion

This present nationwide study evaluated the overall survival of gastric cancer patients aged 75 years and older who were treated with or without neoadjuvant chemotherapy with intent of subsequent curative gastrectomy. The results indicated that patients ≥ 75 years who received neoadjuvant chemotherapy and patients ≥ 75 years directly scheduled for gastrectomy were clinically selected on patient and tumor characteristics and have comparable overall survival on a population-based level.

Furthermore, this study found that a significant proportion of patients ≥ 75 years (15.6%) did not proceed to surgery after receiving neoadjuvant chemotherapy when compared to patients < 75 years (8.9%). In a previous study conducted with data from the Netherlands Cancer Registry between 2006 and 2014, 29.3% of older patients aged ≥ 75 years did not proceed to surgery [12]. Although the percentage of older patients not proceeding to surgery has declined, it still remains high when compared to younger patients.

When our results are compared to landmark randomized controlled trials upon which guidelines are based, in which predominantly younger patients were included, a lower percentage of patients not proceeding to surgery after neoadjuvant chemotherapy is reported, ranging between 3 and 7% [5, 6]. Our study does not fully bring to light why a higher proportion of patients ≥ 75 years did not proceed to surgery compared to patients < 75 years as most of the reasons for not proceeding to surgery are unknown. However, it seems that patients ≥ 75 years did not continue to surgery because of functional decline more often than younger patients, whereas younger patients more often do not proceed to surgery because of disease progression. This is also reflected by the higher proportion of patients ≥ 75 years who did not proceed to surgery that received doublet-based chemotherapy compared to those who did proceed, likely indicative of lower physical fitness at baseline.

In all analyses, patients ≥ 75 years treated with neoadjuvant chemotherapy showed similar overall survival compared to patients ≥ 75 years directly scheduled for surgery. Yet, comparing older patients treated with or without neoadjuvant chemotherapy without randomization, even after statistically correcting for confounders, is particularly difficult when evaluating survival due to selection bias and potentially unknown confounders. This is likely due to that clinicians consider various patient characteristics in the decision-making process for assigning neoadjuvant chemotherapy that cannot be quantified. On the other hand, randomized clinical trials mostly exclude older patients as a result of in- and exclusion criteria [17]. In addition, In case older patients are included in trials, it is probably mainly because they are chronologically old but biologically young [9].

Older patients differ significantly from each other in terms of functional status and number of comorbidities. In this study, older patients directly scheduled for surgery had a higher age, worse WHO performance status, greater ASA score, with clinically less affected lymph nodes, and less often cT3–4 stage tumors, compared to those treated with neoadjuvant chemotherapy. Therefore, it seems that non-fit patients with less advanced disease were directly scheduled for surgery, and more physically fit patients with advanced disease allocated to neoadjuvant chemotherapy. This suggests that clinicians highly select older patients who may benefit from chemotherapy and those who may not, given that the overall survival rates were high in patients ≥ 75 years directly scheduled for surgery and comparable to those treated with neoadjuvant chemotherapy. However, the question of whether the survival of those treated with neoadjuvant chemotherapy would have been worse if they had only received surgery, and vice versa, remains unanswered.

The results of this study showed that the 3-year overall survival rates of patients ≥ 75 years who received FLOT regimen (56%) or anthracycline-based triplet therapy (46%) was similar to the results of the FLOT4 trial (56% in the FLOT group and 48% in the ECF/ECX group) [6]. In the FLOT4 trial, a trend in increased overall survival was found for the subgroup of patients over 70 years old who received FLOT regimen compared to those who received anthracycline-based triplet therapy, but this trend did not achieve statistical significance. Similarly, our data may suggest that older patients treated with FLOT have better overall survival than older patients treated with anthracycline-based triplet therapy, although statistical significance was neither reached in the current study. Furthermore, a higher percentage of patients ≥ 75 years who received FLOT regimen proceeded to surgery compared to older patients who received anthracycline-based triplet regimen, however this comparison was also not statistically significant.

In the group of patients ≥ 75 years who received neoadjuvant chemotherapy, 63% patients completed all neoadjuvant chemotherapy cycles. This percentage is considerably lower compared to the results of the MAGIC [5] (91%) and FLOT [6] (91%) trials, including the subgroup analysis study of the randomized phase II FLOT 65 + trial, in which 18 out of 21 (85%) patients over the age of 65 years with potentially resectable adenocarcinoma of the esophagogastric junction and the stomach completed all four cycles of preoperative FLOT [18]. The reason for not completing all chemotherapy cycles is unknown in the current study. Various reasons can be considered, including toxicity, decreased quality of life and/or reduced functionality, for which older patients are more prone to. Several studies have shown a decrease in physical fitness immediately after neoadjuvant chemotherapy in patients with esophagogastric cancer [19, 20]_._

In line with previous studies on chemotherapy [6, 21–24], no significant difference was found in postoperative complications and length of stay between patients ≥ 75 years treated with or without neoadjuvant chemotherapy. Although, our results showed that patients ≥ 75 years who were directly scheduled for surgery had a greater risk for adverse outcomes postoperatively in terms of mortality compared to patients ≥ 75 years preoperatively treated with chemotherapy (8.3% vs. 3.2%). The highest postoperative mortality rates are shown among patients with comorbidities and among patients ≥ 75 years [10]. In our study, the higher postoperative mortality likely reflects the selection of non-fit patients for a surgery alone approach.

The findings of the current study are of particular importance for patients with borderline fitness, where further loss of physical fitness after neoadjuvant chemotherapy may be crucial, and in whom overall survival may even be improved by directly scheduling for gastrectomy without preoperative treatment. However, it is equally important to exercise caution when considering withholding chemotherapy from older, yet otherwise fit patients. As indicated by the FLOT4 trial, patients older than 70 years seem to benefit from FLOT regimen when carefully selected [6]. This highlights the necessity of selecting older patients for chemotherapy based not solely on their chronological age, but also on their overall health status, including any coexisting medical conditions and physical fitness, thereby considering their biological age. Physical fitness has traditionally been determined using tools of which the score generally varies between clinicians, such as the WHO-performance status [25] or Karnofsky-performance score [26, 27]. A more objective measure of physical fitness, such as cardiopulmonary exercise testing by measuring oxygen uptake, may be of important clinical interest in selecting patients for neoadjuvant treatment, as chronological age alone seems not a contraindication for either preoperative or surgical treatment anymore [28]. Furthermore, a more functional definition of older patients could allow for better comparison in clinical studies.

This study has several limitations. Patients treated with neoadjuvant chemotherapy and diagnosed with non-curable disease during surgery were not excluded from the analysis since they underwent a staging laparoscopy. As the diagnostic sensitivity of staging laparoscopy is not 100%, there may be patients included in this study whose incurable condition was missed during staging laparoscopy [14]. However, excluding these patients would be incorrect as treatment with neoadjuvant chemotherapy does carry the risk of interval metastasis and disease progression [29]. Secondly, it is unknown why patients were directly scheduled for a resection as this information is not registered in the NCR. The reason for not proceeding to surgery was unknown in the majority of patients and therefore we could not fully identify why patients did not proceed to surgery. In addition, missing data in known variables, e.g. WHO performance status, could have led to suboptimal adjustment in the multivariable and sensitivity analyses. Furthermore, the stratified results across age categories, should be critically interpreted, as the number of patients decreased with increasing age category, particularly the group of patients older than 80 years was relatively small. Preferably, we would have performed similar analyses in the group of patients older than 80 years, as the question to whether or not to start with neoadjuvant chemotherapy is especially difficult in this age group. Lastly, the number of older patients decreased when stratified for FLOT regimen and anthracycline-based triplet therapy, thereby reducing the power of the study to detect possible significant results. The question remains whether older patients benefit more from chemotherapy in the current era of FLOT.

In conclusion, the clinical selection of patients ≥ 75 years likely effected comparable overall survival for those treated with or without neoadjuvant chemotherapy. However, the proportion of patients ≥ 75 years who did not proceed to surgery following neoadjuvant chemotherapy was significantly higher when compared to younger patients. Therefore, neoadjuvant chemotherapy should be considered with more caution in older patients with gastric cancer, without withholding those who may benefit from chemotherapy. More research is needed to identify older patients for whom neoadjuvant treatment may preclude a potentially curative resection. Lastly, outcomes in these older patients should be investigated more thoroughly in future randomized studies on perioperative chemotherapy and surgery.

## Supplementary Information

Below is the link to the electronic supplementary material.

Supplementary file 1 (DOCX 18 KB)

## Data Availability

The data supporting this study's findings are not publicly available due to the privacy of research participants. However, the data are available from the corresponding author (MIvBH) upon reasonable request.
