# Supplementary material for: Neoadjuvant chemotherapy in older patients with gastric cancer undergoing surgery: a population-based cohort study
Source: Gastric Cancer. 2023 Jun 7;26(5):763–74. doi: 10.1007/s10120-023-01404-2 (PMC10361849; doi:10.1007/s10120-023-01404-2)
Supplement: Supplementary file 1 — (DOCX 18 KB) [file 10120_2023_1404_MOESM1_ESM.docx]

**Supplementary material.**

**Supplementary Table 1.** Baseline and tumor characteristics of patients ≥75 years in the propensity score matched sample.

|  | **Matched comparison** | |  |
| --- | --- | --- | --- |
| **Patients characteristics** | **Neoadjuvant chemotherapy age ≥75 (N=169)** | **No neoadjuvant chemotherapy age ≥75 (N=169)** | **SMD** |
| Age, median (IQR), years | 78 (76-79) | 78 (76-80) | 0.09 |
| Sex, male no./total no. (%) | 104 (61.5) | 107 (63.3) | 0.04 |
| **WHO performance status, no./total no. (%)** |  |  |  |
| 0 | 46 (27.2) | 52 (30.8) | 0.07 |
| 1 | 57 (33.7) | 53 (31.4) | 0.05 |
| ≥2 | 11 (6.5) | 10 (5.9) | 0.03 |
| Unknown | 55 (32.5) | 54 (32.0) | 0.01 |
| **Number of comorbidity categories^*^, no./total no. (%)** |  |  |  |
| 0 | 61 (36.1) | 63 (37.3) | 0.02 |
| 1 | 56 (33.1) | 61 (36.1) | 0.06 |
| ≥2 | 39 (23.1) | 35 (20.7) | 0.06 |
| Unknown | 13 (7.7) | 10 (5.9) | 0.07 |
| **Tumor location, no./total no. (%)** |  |  |  |
| Proximal | 80 (47.3) | 77 (45.6) | 0.04 |
| Distal | 64 (37.9) | 69 (40.8) | 0.06 |
| Diffuse | 16 (9.5) | 15 (7.7) | 0.06 |
| Unknown | 10 (5.9) | 10 (5.9) | 0.03 |
| **cT-stage, no./total no. (%)** |  |  |  |
| T1-T2 | 78 (46.2) | 75 (44.4) | 0.04 |
| T3-T4a | 65 (38.5) | 70 (41.4) | 0.06 |
| Tx | 26 (15.4) | 24 (14.2) | 0.03 |
| **cN-stage, no./total no. (%)** |  |  |  |
| N0 | 102 (60.4) | 100 (59.2) | 0.02 |
| N1 | 44 (26.0) | 41 (24.3) | 0.04 |
| ≥N2 | 18 (10.7) | 21 (12.4) | 0.05 |
| Nx | 5 (3.0) | 7 (4.1) | 0.07 |
| **Tumor differentiation, no./total no. (%)** |  |  |  |
| Well-moderate | 62 (36.7) | 57 (33.7) | 0.06 |
| Poorly | 83 (49.1) | 89 (52.7) | 0.07 |
| Unknown | 24 (14.2) | 23 (14.2) | 0.02 |
| *WHO* World Health Organization, *ASA* American Society of Anesthesiologists, *IQR* interquartile range, *SMD* standardized mean difference.  ^*^According to Charlson Comorbidity Index. | | | |

**Supplementary Table 2.** Comparison of patients ≥75 years treated with neoadjuvant chemotherapy by FLOT regimen and anthracycline-based triplet therapy.

|  | **EOX/ECC/EOF/ECF (N=119)** | **FLOT (N=77)** | **p-value** |
| --- | --- | --- | --- |
| **Course of neoadjuvant regime no./total no. (%)** |  |  | 0.648 |
| Completed all cycles | 80 (67.2) | 51 (66.2) |  |
| Reduction in cycles | 36 (30.3) | 22 (28.6) |  |
| Unknown | 3 (2.5) | 4 (5.2) |  |
| **Not proceeded to surgery no./total no. (%)** | 15 (12.6) | 5 (6.5) | 0.145 |
|  | **N=15** | **N=5** |  |
| **Reasons for not proceeding to surgery no./total no. (%)** |  |  | 0.182 |
| Non-curable disease after restaging | 4 (26.7) | 0 (0.0) |  |
| Poor functional status | 6 (40.0) | 3 (60.0) |  |
| Patient’s request | 0 (0.0) | 1 (20.0) |  |
| Low tumorload | 0 (0.0) | 0 (0.0) |  |
| Deceased | 0 (0.0) | 0 (0.0) |  |
| Unknown | 5 (33.3) | 1 (20.0) |  |
| *EOX* epirubicine, oxaliplatin, capecitabine, *ECC* epirubicine, cisplatin, capecitabine, *EOF* epirubicine, oxaliplatin, 5-fluorouracil, *ECF* epirubicine, cisplatin, 5-fluorouracil, *FOLFOX* 5-fluorouracil, oxaliplatin, *CAPOX* capecitabine, oxaliplatin, *FLOT* 5-fluorouracil, leucovorin, oxaliplatin, docetaxel, *DOC* docetaxel, oxaliplatin, capecitabine. | | | |

**Supplementary Table 3.** Comparison of baseline characteristics and neoadjuvant treatment regimen of patients ≥75 years who did and did not proceed to surgery after neoadjuvant chemotherapy.

| **Patients characteristics** | **Not proceeded to surgery age ≥75 (N=43)** | **Proceeded to surgery age ≥75 (N=232)** | **p-value** |
| --- | --- | --- | --- |
| Age, median (IQR), years | 77 (76-79) | 77 (76-78) | 0.070 |
| Sex, male no./total no. (%) | 31 (72.1) | 141 (60.8) | 0.159 |
| **WHO performance status, no./total no. (%)** |  |  | 0.076 |
| 0 | 13 (30.2) | 90 (38.8) |  |
| 1 | 13 (30.2) | 83 (35.8) |  |
| ≥2 | 5 (11.6) | 8 (3.4) |  |
| Unknown | 12 (27.9) | 51 (22.0) |  |
| **Number of comorbidity categories*, no./total no. (%)** |  |  | 0.783 |
| 0 | 16 (37.2) | 96 (41.4) |  |
| 1 | 13 (30.2) | 77 (33.2) |  |
| ≥2 | 10 (23.3) | 45 (19.4) |  |
| Unknown | 4 (9.3) | 14 (6.0) |  |
| **Tumor location, no./total no. (%)** |  |  | 0.206 |
| Proximal | 25 (58.1) | 101 (43.5) |  |
| Distal | 10 (23.3) | 89 (38.4) |  |
| Diffuse | 7 (16.3) | 32 (13.8) |  |
| Unknown | 1 (2.3) | 10 (4.3) |  |
| **cT-stage, no./total no. (%)** |  |  | 0.145 |
| T1-T2 | 14 (32.6) | 91 (39.2) |  |
| T3-T4a | 19 (44.2) | 113 (48.7) |  |
| Tx | 10 (23.3) | 28 (12.1) |  |
| **cN-stage, no./total no. (%)** |  |  | 0.027 |
| N0 | 18 (41.9) | 130 (56.0) |  |
| N1 | 11 (25.6) | 69 (29.7) |  |
| ≥N2 | 11 (25.6) | 28 (12.1) |  |
| Nx | 3 (7.0) | 5 (2.2) |  |
| **Tumor differentiation, no./total no. (%)** |  |  | <0.001 |
| Well-moderate | 11 (25.6) | 84 (36.2) |  |
| Poorly | 14 (32.6) | 108 (46.6) |  |
| Undifferentiated | 0 (0.0) | 0 (0.0) |  |
| Unknown | 18 (41.9) | 40 (17.2) |  |
| **Interval between diagnosis and onset of neoadjuvant therapy (days), median (IQR)** | 47 (32-57) | 42 (31-54) | 0.462 |
| **Neoadjuvant chemotherapy regime, no./total no. (%)** |  |  | <0.001 |
| EOX/ECC/EOF/ECF | 15 (34.9) | 104 (44.8) |  |
| FOLFOX/CAPOX | 12 (27.9) | 42 (18.1) |  |
| FLOT | 5 (11.6) | 72 (31.0) |  |
| DOC | 0 (0.0) | 7 (3.0) |  |
| Other | 11 (25.6) | 7 (3.0) |  |
| **Course of neoadjuvant regime no./total no. (%)** |  |  | <0.001 |
| Completed all cycles | 15 (34.9) | 159 (68.5) |  |
| Reduction in cycles | 17 (39.5) | 59 (25.4) |  |
| Unknown | 11 (25.6) | 14 (6.0) |  |
| *IQR* interquartile range, *WHO* World Health Organization, *EOX* epirubicine, oxaliplatin, capecitabine, *ECC* epirubicine, cisplatin, capecitabine, *EOF* epirubicine, oxaliplatin, 5-fluoruracil; *ECF* epirubicine, cisplatin, 5-fluorouracil, *FOLFOX* 5-fluorouracil, oxaliplatin, *CAPOX* capecitabine, oxaliplatin, *FLOT* 5-fluorouracil, leucovorin, oxaliplatin, docetaxel, *DOC* docetaxel, oxaliplatin, capecitabine. | | | |

**Supplementary Table 4.** Overall survival of patients ≥75 years treated with or without neoadjuvant chemotherapy in the propensity score matched sample.

|  | **Neoadjuvant chemotherapy age ≥75 (N=169)** | **No neoadjuvant chemotherapy age ≥75 (N=169)** | **p-value** |
| --- | --- | --- | --- |
| Overall survival (months), median (95% CI) | 36.7 (27.5-45.9) | 28.6 (17.8-39.3) | 0.277 |
| 3-year overall survival % | 52 | 45 |  |
| 5-year overall survival % | 38 | 37 |  |
| Unadjusted hazard ratio (95% CI) | 0.85 (0.64-1.14) | 1 | 0.278 |
| *CI* confidence interval. | | | |
